# Proteomics Parameters for Assessing Authenticity of Grated Grana Padano PDO Cheese: Results from a Three-Year Survey

**DOI:** 10.3390/foods13030355

**Published:** 2024-01-23

**Authors:** Luisa Pellegrino, Veronica Rosi, Marta Sindaco, Paolo D’Incecco

**Affiliations:** Department of Food, Environmental and Nutritional Sciences (DeFENS), Università degli Studi di Milano, Via G. Celoria 2, 20133 Milan, Italy; luisa.pellegrino@unimi.it (L.P.); veronica.rosi@unimi.it (V.R.); marta.sindaco@unimi.it (M.S.)

**Keywords:** cheese authenticity, Grana Padano cheese, grated cheese, proteomics, free amino acids, cheese ripening, capillary zone electrophoresis

## Abstract

Assessing the authenticity of PDO cheeses is an important task because it allows consumer expectations to be fulfilled and guarantees fair competition for manufacturers. A 3-year survey was carried out, analyzing 271 samples of grated Grana Padano (GP) PDO cheese collected on the European market. Previously developed analytical methods based on proteomics approaches were adopted to evaluate the compliance of market samples with selected legal requirements provided by the specification for this cheese. Proteolysis follows highly repeatable pathways in GP cheese due to the usage of raw milk, natural whey starter, and consistent manufacturing and ripening conditions. From selected casein breakdown products, it is possible to calculate the actual cheese age (should be >9 months) and detect the presence of excess rind (should be <18%). Furthermore, due to the characteristic pattern of free amino acids established for GP, distinguishing it from closely related cheese varieties is feasible. Cheese age ranged from 9 to 25 months and was correctly claimed on the label. Based on the amino acid pattern, three samples probably contained defective cheese and there was only one imitation cheese. Few samples (9%) were proven to contain some excess rind. Overall, this survey highlighted that the adopted control parameters can assure the quality of grated GP.

## 1. Introduction

The mislabeling of food products is an increasingly adopted malpractice in the contemporary global market. In most cases, the impact is exclusively of economic nature, such as when a low-value product is intentionally substituted or added to a similar one of higher value. This is the case of foods bearing a Protected Designation of Origin (PDO) recognized by the European Union legislation [1]. Dedicated analytical tools are required to tackle the mislabeling of PDO foods and to assure consumers they are not buying incorrectly labeled products [2,3]. In general, PDO foods shall be entirely produced in a defined geographical area and following a traditional manufacturing process described in the product specification. Compliance with these provisions ensures the superior quality and unique sensorial characteristics of a PDO food and justifies the higher price they command compared to similar generic products. In this context, quality control schemes, including regular monitoring systems and effective controls, should be adopted to guarantee the compliance of the marketed products with their product specification. The authentication of the geographical origin of a cheese can be achieved by various approaches mostly based on modern analytical techniques [2,4]. Mass spectrometry determination of stable isotope ratios and trace elements [5,6], DNA-based identification of cheese microbiota [7,8], and untargeted metabolomics evaluating several cheese components by LC-MS [9], Raman spectroscopy [10] or by ^1^H NMR [11] are among the most successfully investigated approaches. However, the inspection of other quality characteristics of PDO cheeses, especially those relevant in the grated product, such as the ripening time or the presence of excess rind, requires targeted analytical methods that enable the quantification of known analytes within the complex cheese matrix with high sensitivity and accuracy. Proteomics is a powerful tool for characterizing high-protein foods such as cheese [12,13,14]. In previous studies we have demonstrated that proteolysis proceeds throughout highly repeatable pathways in PDO raw-milk hard cheeses, such as Grana Padano (GP) and Parmigiano Reggiano, due to the controlled manufacturing conditions [15,16]. Proteolysis begins with chymosin and plasmin activities on casein, while further progress follows the dynamics of microbial species in raw milk and those added with the natural whey starter [17,18,19,20]. These species progressively release a pool of proteolytic enzymes that actively breakdown casein during a ripening period that may last up to 2–3 years. Upon interactions between enzymes and substrates, which both evolve over time, casein breakdown follows typical pathways and the released products, i.e., peptides and free amino acids, accumulate at a relative constant rate. This made it possible to build fingerprints of these two cheeses. Early studies evaluated the progress of casein degradation in GP cheese by calculating the ƴ-casein/β-casein ratio determined by urea–polyacrylamide gel electrophoresis. The ratio proved to increase linearly during cheese ripening up to 15 months [21]. Similarly, the ratios between selected peptides deriving from α_s1_-casein and the parent protein were reported to increase in GP cheese during ripening, with values differing (*p* < 0.01) between cheeses aged 4–9 months and those aged 12 months [22]. Using large databases of analytical data, we developed specific algorithms controllable at each ripening time that allow the authenticity of GP cheese to be assessed, which is particularly useful when the cheese is sold in grated form and hence its identity is no longer evident. The obtained parameters proved to reliably identify the presence of imitation cheeses, to calculate the actual cheese age and the presence of excess rind in the grated cheese [15,16]. In this study, we present outcomes of the application of these parameters in assessing the authenticity of 271 samples of grated GP cheese collected on the European marketplace within a three-year survey. The significance of the multi-parameter approach in identifying various frauds is discussed. Implementation of quality control schemes for grated PDO GP allows consumers’ expectations to be fulfilled and guarantees fair competition for producers. This study will be also helpful in providing new insight into the use of proteomics in cheese authenticity analysis. 

## 2. Materials and Methods

### 2.1. Cheese Samples

A total of 271 commercial samples of grated Grana Padano PDO cheese (each in 100 g or 150 g packages) were collected randomly at a large number of supermarkets and groceries in 17 different EU countries, as detailed in Table S1. The survey covered three years, from January 2020 to December 2022. During this period, cheese samples were brought directly to the laboratory under refrigerated conditions and analyzed within a week and before the expiry date. Cheese from each package was grated further before analyses in order to achieve uniform granulometry. Cheese samples were analyzed for free amino acid patterns to assess authenticity and for casein and peptide patterns to evaluate the cheese age and detect the presence of excess rind.

### 2.2. Determination of Free Amino Acids 

A 2.5 g aliquot of cheese was added to 40 mL of Na citrate buffer in a beaker, kept under magnetic stirring for 15 min and then carefully homogenized with an Ultra-Turrax (5 min at 11,000 rpm). The extract was filtered through a paper filter and 10 mL of the filtrate was transferred into a 25 mL volumetric flask, with 10 mL of 7.5% (*w*/*v*) 5-sulphosalicylic acid (pH 1.7–1.8) added dropwise under stirring, diluted to the volume mark with Na citrate buffer, and filtered. Finally, 10 mL of filtrate was transferred into a 100 mL volumetric flask, with norleucine added as an internal standard, made up to the volume mark with 0.2 N lithium citrate pH 2.2, and filtered through a 0.2 µm regenerated cellulose disposable filter (Merck, Milan, IT) prior to injection. The free amino acid (FAA) content was determined by ion exchange chromatography (IEC) according to a previously described method [23]. A Biochrom 30+ amino acid analyzer (Erreci, Milan, Italy) was used. Injection volume was 100 µL. The quantification was carried out by the external calibration method and data were expressed on a cheese protein basis and as a relative percentage. Cheese protein content was determined using the Kjeldahl method [24]. Analyses were conducted in duplicate. The mean relative content of each FAA was compared with the respective reference value established by the Consortium of GP cheese [25] on a set of more than 300 samples of authentic cheeses ripened for from 9 to 32 months. The Z-scores were calculated for the actual content of each FAA and the quality control scheme adopted by the Consortium was applied to evaluate their acceptability [23].

### 2.3. Casein and Peptide Analysis by Capillary Zone Electrophoresis (CZE) 

The analytical conditions of the CZE method described in a previous study [16] were adopted. Briefly, 1 g of grated cheese was added to 10 mL of sample buffer in a glass vial and gently dispersed for 4 h. The sample was then diluted 5 fold with the same buffer and filtered through a 0.22 µm membrane (PVDF, Millipore, Milan, Italy). The separation was carried out on a hydrophilically coated capillary column (DB-WAX 126-7012, J&W Agilent Technologies, Milan, Italy) with the following characteristics: 50 µm i.d., 0.05 µm coating, 500 mm effective length, 100 × 800 µm slit opening. A P/ACE^TM^ MDQplus capillary electrophoresis equipment (AB Sciex, Milan, Italy), including a UV detector set at 214 nm, was used. Analyses were carried out in duplicate.

The cheese age (in months) was calculated using the equation defined in a previous study [16]:

Age (months) = 0.91 × [(100 × pγ_3_–CnA)/(pγ_3_–CnA + γ_3_–CnA)] + 4.33 (1)

where pγ_3_–CnA is the corrected peak area of the fraction derived from cyclization of the N-terminal Glu of γ_3_–CnA.

The content of cheese rind was evaluated using the equation previously defined [15] and expressed as Rαs value:

Rαs = (α_s2′_–Cn/α_s1_–PL) × 100 (2)

where α_s2′_–Cn is the corrected peak area of α_s2′_–Cn and α_s1_–PL is the corrected peak area of α_s1_–Cn (f80–199).

A value of Rαs > 7 indicates that an excess of rind is present in the grated cheese [15].

### 2.4. Statistical Treatment of Data

Statistical treatment of data was carried out using the SPSS Win 12.0 software, version 27 (SPSS, IBM Corp., Chicago, IL, USA).

## 3. Results

### 3.1. Outline of the Survey

The samples of grated GP cheese considered in the survey were assessed for requisites provided by specification [25] that are not directly (visually) inspectable in the packaged retail products: (i) cheese authenticity and genuineness, i.e., matching with the reference free amino acid (FAA) pattern of GP; (ii) cheese age, i.e., matching with the minimum ripening time of 9 months or with the age indicated on the package; (iii) presence of excess rind, i.e., matching with the maximum amount of rind of 18%. 

### 3.2. Cheese Authenticity and Genuineness Assessment

**Figure 1:**
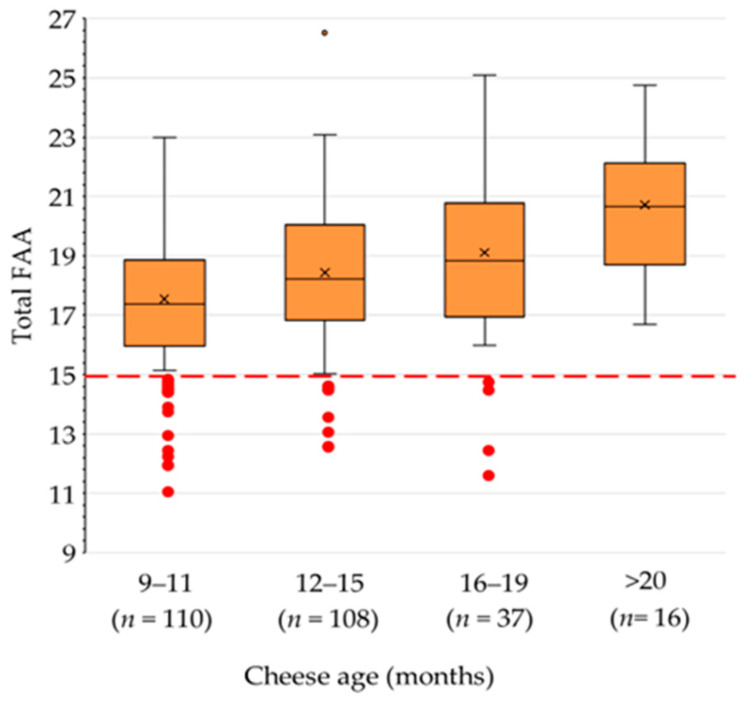
Free amino acid (FAA) content (% cheese protein) in 271 commercial samples of grated Grana Padano cheese of different ages. The number in brackets is the number of samples in each age range. Data points in red are individual samples with an FAA content lower than the minimum threshold of 15%.

The free amino acid (FAA) composition of the 271 cheese samples is reported in Table S2. The total content of FAA roughly ranged from 11 to 26% on a cheese protein basis (Figure 1). Such a wide range of values primarily depends on the different age of the cheeses that were analyzed, i.e., from 9 to 32 months. According to the product specification, GP should be ripened for 9 months at least. Nevertheless, grated cheese can be prepared from longer-ripened cheeses. This aspect is discussed later. The average content of FAA clearly increased with increasing cheese age, although the variability was large. Although there is no regulatory minimum limit for the total content of FAA in grated GP cheese, previous studies considered that the value (+2 standard deviation, SD) should not be lower than 15% on a cheese protein basis [15]. A total of 14 samples (5%) of this survey did not reach this threshold, having FAA values barely compatible with the respective age.

**Figure 2:**
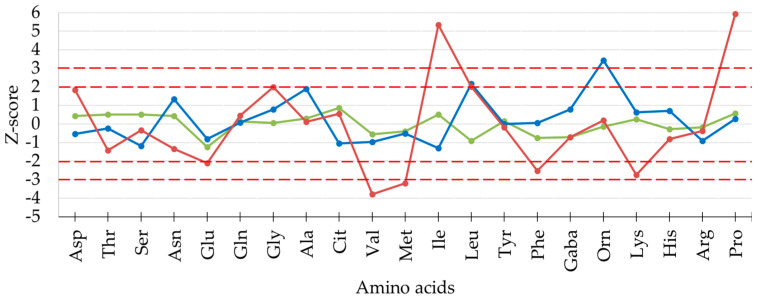
Evaluation of the content of individual free amino acids by Z-score values for three distinct samples of grated GP cheese in the present study. Green and blue lines refer to GP cheeses complying with established Z-score limits. Red line refers to a sample not complying with established Z-score limits.

The true FAA composition of GP was established in a previous study by considering a set of 260 samples of known origin and age [15] and subsequently consolidated by analyzing further samples [23]. The compliance of the FAA composition of commercial samples of the present survey was evaluated by calculating the Z-score (number of SD) for each amino acid, i.e., the difference between the actual content and the content expected in GP cheese. The quality control scheme states that for GP cheese, a maximum of 4 FAAs can have a Z-score > 2.0 and, among these, only one can have a Z-score > 3.0 [23]. Examples of samples complying and not complying with these limits are shown in Figure 2. 

**Figure 3:**
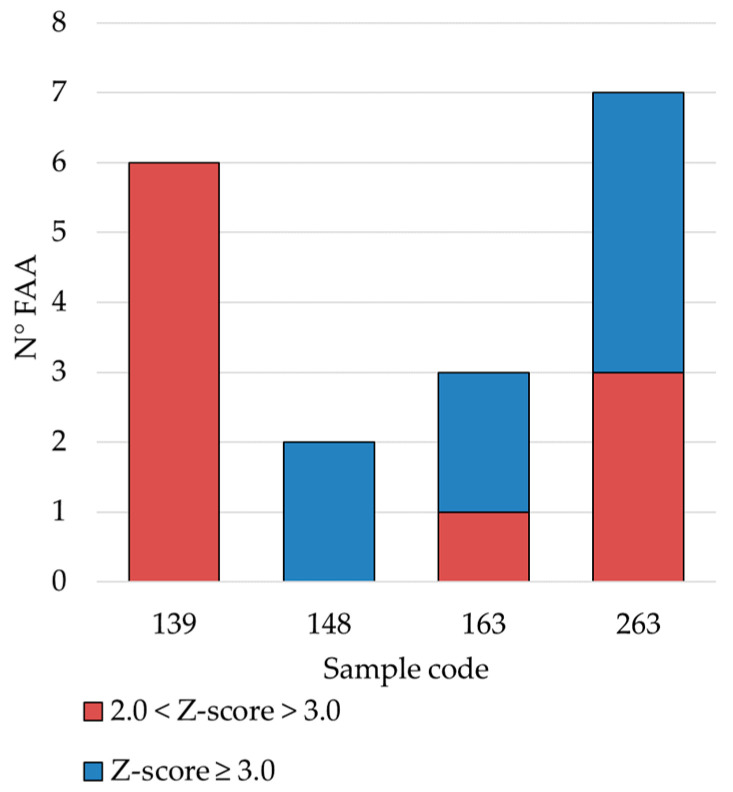
Numbers of free amino acids (FAAs) having a Z-score value between 2 and 3 or higher than 3 in four distinct samples of grated GP cheese in the present study. For sample codes, refer to Table S1.

It was observed that Z-scores were <2.0 for all FAAs in 183 samples (67%) and were between 2.0 and 3.0 for one or two amino acids in 82 samples (30%), with values that can be considered as due to the natural variability. In contrast, in four samples, Z-scores exceeded the limits defined above (Figure 3). Indeed, these samples proved to have deviating content for up to seven different FAAs, indicating that their ripening process did not follow the typical pathways of GP cheese. Interestingly, the FAAs that showed highest absolute values of Z-score were ƴ-aminobutyric acid, ornithine, and histidine. 

### 3.3. Determination of Cheese Age

The method previously developed for calculating the actual age of GP cheese [16] was used for evaluating the samples in this study. The method had an error of ±1.5 months. All samples reached the minimum ripening time and thus conformed. Around one-third of samples (91 samples) were produced from cheeses aged for 9–11 months, with the remaining samples ripened for between 12 and 25 months, with the notable exception of one sample ripened for 32 months. For 26 of the cheeses ripened for the longest, the manufacturers indicated the age on the label and thus it was possible to verify the accuracy of the information given to consumers. Labeled and calculated cheese ages are compared in Table 1. All samples proved to be obtained from cheeses that globally had an age equal to or higher than that stated on the package.

### 3.4. Determination of Cheese Rind Content

**Figure 4:**
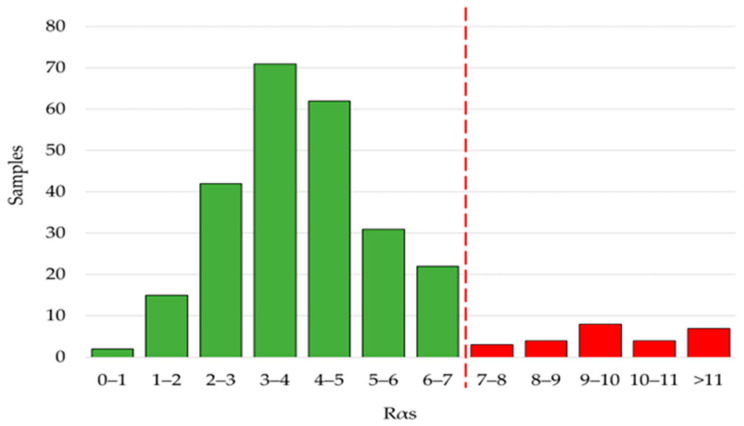
Values of Rαs determined in 271 commercial samples of Grana Padano cheese. Samples with values of Rαs ≤ 7 are regular (green bars). Samples with values of Rαs > 7 are irregular (red bars) since they contain more than 18% rind.

A crucial aspect of the quality control of grated cheese is the presence of excess rind. The product specification of GP indicates that the content of rind in the grated cheese should not exceed 18% by weight. Based on a proteomic approach that considers specific peptides characteristic of the rind, we had proposed the parameter Rαs that would correlate well with the content of rind in grated GP cheese, regardless of its age [15]. According to this approach, when the content of rind is 18%, the Rαs value can be up to 7 at +3 SD confidence level. The Rαs values of the samples of the present survey are shown in Figure 4. The majority of the analyzed samples (91%) had Rαs values below the threshold of 7, with the maximum frequency at 3–4 and a natural variability due to the fact that the amount of rind in the cheese wheel is typically lower than the maximum permitted. Some samples fell close to the threshold value, indicating that the amount of rind slightly exceeded the maximum level or that, after the grating operation, the rind was not uniformly distributed within the grated cheese by accurate mixing. Conversely, 26 samples (9%) proved to contain excess rind in various amounts, with 1 sample having a value of Rαs up to 21. 

## 4. Discussion

PDO cheeses are appreciated by the consumer for their traditional manufacturing and link to a specific geographical area. In addition to the measures adopted to achieve the traceability of the entire manufacturing process [25], to guarantee consumer trust when buying grated GP cheese, a set of targeted methods was developed to verify compliance with specifications of the products on the market. The present 3-year survey focused on the evaluation of authenticity of the grated cheese, the actual age, and the possible addition of excess rind. 

The FAA profile was used to discriminate cheeses that did not undergo a typical proteolytic process. Deviation from the typical profile of grated GP could be due to the addition of either imitation cheeses [15] or being cheeses that develop defects such as holes or cracks [26] and therefore are no longer suitable for selling as portions. Both these types of products are explicitly not permitted in grated GP cheese. As FAAs are principally released from casein by microbial proteases during cheese ripening, lactic acid bacteria species typically colonizing this cheese are responsible for the highly repeatable FAA profile [18,19]. Consequently, the presence of foreign or contaminating species can easily alter this profile. The inoculation of adjunct cultures is rather common in industrial cheese manufacture to accelerate the ripening process; however, it has an impact on FAA composition [27]. Correlations existing between cheese microbiota and specific FAAs have been highlighted by several authors [28,29,30]. In a previous study, it was demonstrated that in GP cheeses intentionally contaminated with *Clostridium tyrobutyricum*, the main species responsible for the so called “late blowing” defect in hard cheeses, high contents of free ƴ-aminobutyric acid and ornithine were produced by catabolism of glutamic acid and arginine, respectively [31]. Herein, three of the analyzed samples showed a similarly anomalous pattern, evidenced by the high Z-scores (as absolute values) of the involved amino acids; thus, they were most probably obtained from defective cheeses. Indeed, the gas produced by Clostridia upon the initial development of the late blowing may originate from holes or cracks so small that they are not detected during the cheese inspection preceding the grating step [26]. Furthermore, one sample had an extremely anomalous profile of FAAs, with Z-scores in the range 2.0–3.0 for three amino acids (glutamic acid, phenylalanine, and lysine) and Z-scores > 3.0 for other four (valine, leucine, isoleucine, and proline). These amino acids are derived from protease activities; none are directly correlated to cheese defects or are derive from metabolic pathways of microorganisms. This situation suggests that this grated cheese is not authentic GP. It was previously demonstrated that deviations from the traditional manufacturing process of GP, such as the pasteurization or centrifugation of milk, the usage of selected starters or adjunct cultures, and mechanized curd processing, all have an impact on the final release of FAAs [15,32,33]. Indeed, the discriminating power considering the whole FAA profile is that each cheese is evaluated on the basis of 21 variables at the same time. Furthermore, the same sample barely reached a minimal ripening period of 9 months, had an unacceptably low content of FAA (13.3 g/100 g protein), and proved to contain excess rind (Rαs = 12.2). 

In grated cheeses, poor mixing after industrial grating can be an additional source of variability, since FAA are not uniformly distributed within the wheel [15,34]. Low water activity in the rind of extra-hard cheeses impedes proteolysis, causing the FAA content in GP cheese rind (4–6 mm thick) to be lower than 8–10 g/100 g protein, regardless the ripening period. This explains the weak negative correlation between Rαs value and FAA content in grated GP samples (Figure S1). 

The peptide fraction also evolves throughout typical pathways during the ripening period of GP cheese. Consistently, it was demonstrated that changes in the manufacturing process, such as milk pasteurization or de-bacterization by centrifugation or the usage of microbial coagulant, may affect these pathways in different ways [35,36]. The relevance of peptide fraction as a quality parameter of cheese ripening has been also highlighted in Parmigiano Reggiano cheese [16,37]. When assessing the compliance of marketed grated GP cheese with product specification, the most relevant aspects are the determination of actual cheese age and the detection of the possible presence of excess rind. 

As mentioned before, a minimum ripening time of 9 months is specified for GP cheese. However, manufacturers can produce grated cheese from cheeses aged beyond 9 months and claim the age on the package to increase the value their product. In particular, for cheeses that are ripened over 12 months, the actual age can be indicated on the label. Alternatively, when the ripening time exceeds 20 months, the cheese can be labeled as “Riserva” [25]. It should be mentioned that many cheeses, not necessarily all of the same age, can be grated in the same batch of GP, mostly depending on the size of the cheese factory. Therefore, it is important to assess whether the overall age of the cheese in a single package really corresponds to the age indicated on the label. Indeed, all samples analyzed in this survey proved to be properly ripened and labeled. In a recent survey of grated Parmesan cheese manufactured and sold in Brazil, the ripening time was found to be the most common not complying parameter, even though a minimum ripening time of 6 months is provided in the country [38]. The cheese age was calculated on the basis of the ƴ-casein/β-casein ratio proposed by other authors [21]. 

Based on the specification of the size (diameter and height) of the GP cheese wheel [25], the rind (4–8 mm) can represent up to 18% of the whole weight. The same limit is specified for Parmigiano Reggiano PDO cheese [39]. Excess rind was detected in 26 samples, although Rαs values were sometimes only slightly above the threshold of 7. In these last cases, poor accuracy in grated cheese mixing before filling the packages can be hypothesized. Indeed, due to the lower water content in the outermost part of the cheese [26,30], grated rind normally contains fibrous particles that tend to separate from the rest of the mix. In a recent study of grated Parmigiano Reggiano cheese, the effect of using different grater types, i.e., drum grater or knife mill, was evaluated [40]. Although the cheese grating was conducted on a laboratory scale, the authors observed a higher presence of fibrils in the cheese obtained from drum grating. However, regardless of the type of grater in use, manufacturers should adopt appropriate measures to ensure product homogeneity within the batch.

Overall, 30 samples out of 271 did not comply with 1 or 2 parameters relevant in the specification of GP cheese, but only 1 sample was irregular for the 3 parameters tested. The presence of excess rind was the most frequently detected irregularity. For all samples, the correct age was indicated. 

## 5. Conclusions

The results of this study demonstrate that the noncompliance of grated Grana Padano cheese is rather episodic and may sometimes be unintentional. Indeed, excess rind detected in 9% of samples was mostly at low levels and thus could be due to inaccurate mixing of the product. Only 1 sample out of 271 was irregular for the 3 parameters tested. The challenge of maintaining the integrity and genuineness of this PDO cheese can be achieved by adopting a quality control scheme relying on analytical parameters targeted towards the aspects that are more vulnerable to fraud and manipulation. Mislabeled grated GP cheese can be detected and selectively recognized whenever excess rind, defective cheese, or cheeses other than GP is added. Furthermore, the actual cheese age can be determined and can be used for monitoring cheese ripening and to validate the age indicated on the package. By integrating datasets obtained with different proteomics approaches, the robustness of the quality control scheme here applied increases and provides more confidence in the estimates. This survey provides evidence that the authenticity and quality of grated GP cheese are regularly controlled with regards to the considered parameters. Implementing dedicated tools to perform analytical control of grated PDO cheeses on the market is the best guarantee for the consumers. Furthermore, the analytical approaches adopted in this study represent routinely applicable tools to support the manufacturers in adopting in-house measures to improve the quality of their product. 

## Figures and Tables

**Table 1:** Comparison between the cheese age indicated on the package and that calculated for 26 samples of grated GP cheese in the present study.

Number of Samples	Labeled Cheese Age	Calculated Cheese Age (Months)
3	Over 12 months	12–17
5	14 months	14–19
1	15 months	20
6	Over 16 months	16–21
11	Over 20 months	19–32

## Data Availability

Data is contained within the article.

## Supplementary Materials

The following supporting information can be downloaded at: https://www.mdpi.com/article/10.3390/foods13030355/s1, Figure S1: Correlation of Rαs value with FAA content in grated GP cheese samples; Table S1: Samples in the study; Table S2: Free amino acid composition (% cheese protein) of grated GP cheese samples.

## References

[1] (2012). EU Regulation No. 1151/2012 of the European Parliament and of the Council of 21 November 2012 on Quality Schemes for Agricultural Products and Foodstuffs. Off. J. Eur. Union.

[2] Cardin M., Cardazzo B., Mounier J., Novelli E., Coton M., Coton E. (2022). Authenticity and Typicity of Traditional Cheeses: A Review on Geographical Origin Authentication Methods. Foods.

[3] Menozzi D., Yeh C.H., Cozzi E., Arfini F. (2022). Consumer Preferences for Cheese Products with Quality Labels: The Case of Parmigiano Reggiano and Comté. Animals.

[4] Abedini A., Salimi M., Mazaheri Y., Sadighara P., Sani M.A., Assadpour E., Jafari S.M. (2023). Assessment of cheese frauds, and relevant detection methods: A systematic review. Food Chem..

[5] Camin F., Bertoldi D., Santato A., Bontempo L., Perini M., Ziller L., Stroppa A., Larcher R. (2015). Validation of Methods for H, C, N and S Stable Isotopes and Elemental Analysis of Cheese: Results of an International Collaborative Study: Validation of Methods for Isotopic and Elemental Analysis of Cheese. Rapid Commun. Mass Spectrom..

[6] Pianezze S., Bontempo L., Perini M., Tonon A., Ziller L., Franceschi P., Camin F. (2020). δ34S for tracing the origin of cheese and detecting its authenticity. J. Mass Spectrom..

[7] Gobbetti M., Di Cagno R., Calasso M., Neviani E., Fox P.F., De Angelis M. (2018). Drivers That Establish and Assembly the Lactic Acid Bacteria Biota in Cheeses. Trends Food Sci. Technol..

[8] Guo X., Yu Z., Zhao F., Sun Z., Kwok L.-Y., Li S. (2021). Both Sampling Seasonality and Geographic Origin Contribute Significantly to Variations in Raw Milk Microbiota, but Sampling Seasonality Is the More Determining Factor. J. Dairy Sci..

[9] Rocchetti G., Lucini L., Gallo A., Masoero F., Trevisan M., Giuberti G. (2018). Untargeted metabolomics reveals differences in chemical fingerprints between PDO and non-PDO Grana Padano cheeses. Food Res. Int..

[10] Stocco G., Gomez Gomez-Mascaraque L., Deshwal G.K., Sanchez J.C., Molle A., Pizzamiglio V., Berzaghi P., Gergov G., Cipolat-Gotet C. (2024). Exploring the use of NIR and Raman spectroscopy for the prediction of quality traits in PDO cheeses. Front. Nutr..

[11] Balthazar C.F., Guimarães J.T., Rocha R.S., Pimentel T.C., Neto R.P.C., Tavares M.I.B., Graça J.S., Alves Filho E.G., Freitas M.Q., Esmerino E.A. (2021). Nuclear Magnetic Resonance as an Analytical Tool for Monitoring the Quality and Authenticity of Dairy Foods. Trends Food Sci. Technol..

[12] Gagnaire V., Jardin J., Jan G., Lortal S. (2009). Invited review: Proteomics of milk and bacteria used in fermented dairy products: From qualitative to quantitative advances. J. Dairy Sci..

[13] Pillidge C., Afshari R., Gill H. (2022). Cheese quality and authenticity: New technologies help solve an age-old problem. Microbiol. Aust..

[14] Kritikou A.S., Aalizadeh R., Damalas D.E., Barla I.V., Baessmann C., Thomaidis N.S. (2022). MALDI-TOF-MS integrated workflow for food authenticity investigations: An untargeted protein-based approach for rapid detection of PDO feta cheese adulteration. Food Chem..

[15] Cattaneo S., Hogenboom J.A., Masotti F., Rosi V., Pellegrino L., Resmini P. (2008). Grated Grana Padano cheese: New hints on how to control quality and recognize imitations. Dairy Sci. Technol..

[16] D’Incecco P., Limbo S., Hogenboom J., Rosi V., Gobbi S., Pellegrino L. (2020). Impact of extending hard-cheese ripening: A multiparameter characterization of Parmigiano Reggiano cheese ripened up to 50 months. Foods.

[17] Ferranti P., Itolli E., Barone F., Malorni A., Garro G., Laezza P., Chianese L., Migliaccio F., Stingo V., Addeo F. (1997). Combined high resolution chromatographic techniques (FPLC and HPLC) and mass spectrometry-based identification of peptides and proteins in Grana Padano cheese. Lait.

[18] Gatti M., Bottari B., Lazzi C., Neviani E., Mucchetti G. (2014). Invited review: Microbial evolution in raw-milk, long-ripened cheeses produced using undefined natural whey starters. J. Dairy Sci..

[19] Giraffa G. (2021). The microbiota of Grana Padano cheese. A review. Foods.

[20] Corrigan B.M., Kilcawley K.N., Sheehan J.J. (2021). Validation of a reversed-phase high-performance liquid chromatographic method for the quantification of primary proteolysis during cheese maturation. Int. J. Dairy Technol..

[21] Mayer H.K., Rockenbauer C., Mlcak H. (1998). Evaluation of proteolysis in Parmesan cheese using electrophoresis and HPLC. Lait.

[22] Gaiaschi A., Beretta B., Poiesi C., Conti A., Giuffrida M.G., Galli C.L., Restani P. (2000). Proteolysis of αs-casein as a marker of Grana Padano cheese ripening. J. Dairy Sci..

[23] Hogenboom J.A., D’Incecco P., Fuselli F., Pellegrino L. (2017). Ion-exchange chromatographic method for the determination of the free amino acid composition of cheese and other dairy products: An interlaboratory validation study. Food Anal. Methods.

[24] (2014). Milk and Milk Products—Determination of Nitrogen Content.

[25] Grana Padano (2022). Grana Padano PDO Specification. https://granapadano.kleecks-cdn.com/wp-content/uploads/2023/02/SpecificationsGBOct2022-50252.pdf.

[26] Carminati D., Bonvini B., Rossetti L., Mariut M., Zago M., Giraffa G. (2024). Identification and characterization of the microbial agent responsible of an alteration in spoiled, Grana Padano cheese during ripening. Food Control.

[27] Khattab A.R., Guirguis H.A., Tawfik S.M., Farag M.A. (2019). Cheese ripening: A review on modern technologies towards flavor enhancement, process acceleration and improved quality assessment. Trends Food Sci. Technol..

[28] Afshari R., Pillidge C.J., Dias D.A., Osborn A.M., Gill H. (2020). Microbiota and metabolite profiling combined with integrative analysis for differentiating cheeses of varying ripening ages. Front. Microbiol..

[29] Garbowska M., Pluta A., Berthold-Pluta A. (2020). Contents of functionally bioactive peptides, free amino acids, and biogenic amines in Dutch-Type cheese models produced with different lactobacilli. Molecules.

[30] Araújo-Rodrigues H., Martins A.P., Tavaria F.K., Santos M.T.G., Carvalho M.J., Dias J., Alvarenga N.B., Pintado M.E. (2022). Organoleptic chemical markers of Serpa PDO cheese specificity. Foods.

[31] D’Incecco P., Pellegrino L., Hogenboom J.A., Cocconcelli P.S., Bassi D. (2018). The late blowing defect of hard cheeses: Behaviour of cells and spores of *Clostridium tyrobutyricum* throughout the cheese manufacturing and ripening. LWT.

[32] D’Incecco P., Bettera L., Bancalari E., Rosi V., Sindaco M., Gobbi S., Candotti P., Nazzicari N., Limbo S., Gatti M. (2023). High-speed cold centrifugation of milk modifies the microbiota, the ripening process and the sensory characteristics of raw-milk hard cheeses. Food Res. Int..

[33] Bettera L., Dreier M., Schmidt R.S., Gatti M., Berthoud H., Bachmann H.P. (2023). Selective enrichment of the raw milk microbiota in cheese production: Concept of a natural adjunct milk culture. Front. Microbiol..

[34] De Angelis Curtis S., Curini R., Delfini M., Brosio E., D’ascenzo F., Bocca B. (2000). Amino acid profile in the ripening of Grana Padano cheese: A NMR study. Food Chem..

[35] Franceschi P., Malacarne M., Bortolazzo E., Coloretti F., Formaggioni P., Garavaldi A., Musi V., Summer A. (2022). Automatic milking systems in the production of Parmigiano Reggiano cheese: Effects on the milk quality and on cheese characteristics. Agriculture.

[36] D’Incecco P., Hogenboom J.A., Rosi V., Cabassi G., Pellegrino L. (2022). Effects of microbial coagulants from *Rhyzomucor miehei* on composition, sensory and textural characteristics of long-ripened hard cheeses. Food Chem..

[37] Bottari B., Levante A., Bancalari E., Sforza S., Bottesini C., Prandi B., De Filippis F., Ercolini D., Nocetti M., Gatti M. (2020). The interrelationship between microbiota and peptides during ripening as a driver for Parmigiano Reggiano cheese quality. Front. Microbiol..

[38] Fagnani R., Damião B.C.M., Trentin R.P.S., Zanoni A.P.K. (2022). Authenticity under threat: Grated Parmesan cheese sold in Brazil. J. Dairy Res..

[39] Alinovi M., Mucchetti G., Tidona F. (2019). Application of NIR spectroscopy and image analysis for the characterisation of grated Parmigiano-Reggiano cheese. Int. Dairy J..

[40] Calvini R., Michelini S., Pizzamiglio V., Foca G., Ulrici A. (2022). Evaluation of the effect of factors related to preparation and composition of grated Parmigiano Reggiano cheese using NIR hyperspectral imaging. Food Control.

