# Supplementary material for: Proteomics Parameters for Assessing Authenticity of Grated Grana Padano PDO Cheese: Results from a Three-Year Survey
Source: Foods. 2024 Jan 23;13(3):355. doi: 10.3390/foods13030355 (PMC10855795; doi:10.3390/foods13030355)
Supplement: Supplementary file 1 [file foods-13-00355-s001.zip › Table S1 - Countries of the survey.pdf]

Table S1: EU countries where commercial samples of grated Grana Padano PDO cheese were collected during the three-year survey.

2020 (n = 86)		2021 (n = 78)		2022 (n = 107)	
ITALY	59	ITALY	38	ITALY	33
AUSTRIA	2	BELGIUM	1	BELGIUM	2
BELGIUM	2	SWITZERLAND	4	SPAIN	1
CROATIA	2	UNITED KINGDOM	4	SWITZERLAND	3
DENMARK	1	FRANCE	8	UNITED KINGDOM	2
FRANCE	4	ROMANIA	2	FRANCE	22
GERMANY	5	THE NETHERLANDS	1	ROMANIA	3
CZECH REPUBLIC	2	GERMANY	9	THE NETHERLANDS	1
ROMANIA	3	SLOVENIA	1	GERMANY	18
SLOVENIA	2	AUSTRIA	1	SLOVENIA	4
SPAIN	2	DENMARK	2	AUSTRIA	7
UNITED KINGDOM	2	POLAND	2	DENMARK	1
		CZECH REPUBLIC	2	POLAND	3
		CROATIA	2	CZECH REPUBLIC	3
		FINLAND	1	CROATIA	1
				ESTONIA	1

FINLAND	1
LITHUANIA	1
