# Supplementary figures and images for: Proteomics Parameters for Assessing Authenticity of Grated Grana Padano PDO Cheese: Results from a Three-Year Survey
Source: Foods. 2024 Jan 23;13(3):355. doi: 10.3390/foods13030355 (PMC10855795; doi:10.3390/foods13030355)

Figure S1: Correlation of Ras value with FAA content in grated GP cheese samples.

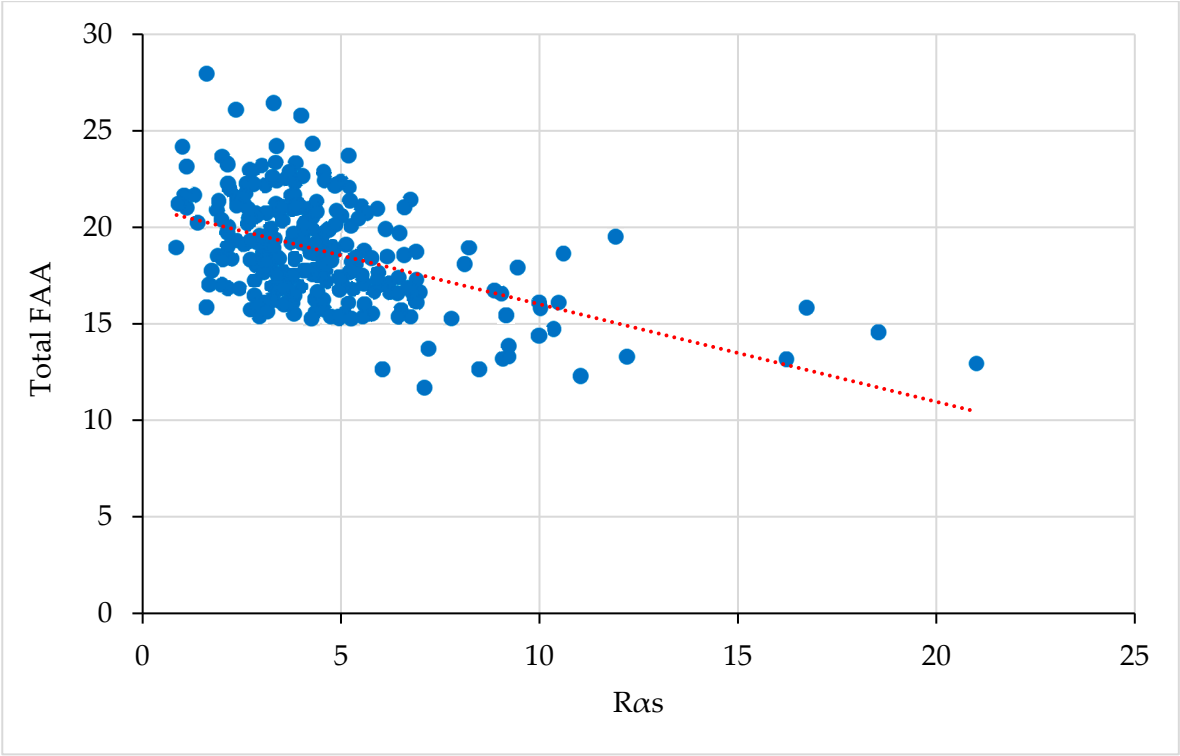

Supplement: Supplementary file 1 [file foods-13-00355-s001.zip › Figure S1. Correlation of Ras value with FAA content in grated GP cheese samples..pdf]
